# Double the efficacy, half the effort: columnar transparent cap-assisted cholangioscopy for bile duct stone extraction

**DOI:** 10.1055/a-2665-7090

**Published:** 2025-08-22

**Authors:** Shan-Shan Hu, Xiao-Gang Liu, Yun-Chao Yang, Jie Hou, Wei-Hui Liu

**Affiliations:** 1 89669 Department of Gastroenterology and Hepatology, Sichuan Provincial Peopleʼs Hospital, School of Medicine, University of Electronic Science and Technology of China, Chengdu, China

In recent years, transparent cap-assisted endoscopy has gained widespread adoption. By stabilising the visual field and expanding the viewing range 1 2 3 4 , it has become extensively utilised in the diagnosis and treatment of digestive tract disorders. Inspired by this technique, we applied transparent cap-assisted cholangioscopy during laser lithotripsy and stone extraction in a case of bile duct stones. The procedure was performed smoothly, achieving highly efficient clinical outcomes with reduced operative effort.

A female patient presented with multiple common bile duct stones. Under clear vision of a cholangioscope fitted with a transparent cylindrical cap ( Fig. 1 ), a hard huge stone obstructing the common bile duct was promptly identified. The transparent cap facilitated accurate assessment of the stone’s size, confirming its relatively large dimensions and necessitating fragmentation prior to piecemeal extraction ( Fig. 2 ). Laser lithotripsy was subsequently performed, with the transparent cap maintaining optimal endoscopic visibility while ensuring stable contact with the stone and providing adequate working space for the laser fibre ( Fig. 3 ). The cap was vertically apposed and stabilised against the stone surface, achieving ideal angulation and stability for effective lithotripsy. Following fragmentation ( Fig. 4 ), the transparent cap enabled aspiration of residual stone fragments that were inaccessible to basket retrieval ( Fig. 5 ), thereby ensuring complete stone clearance ( Video 1 ).

**Fig. 1:**
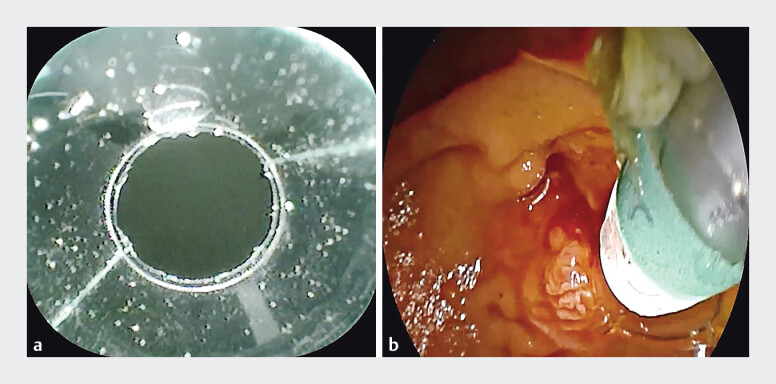
The columnar transparent cap is affixed to the distal end of the choledochoscope.

**Fig. 2:**
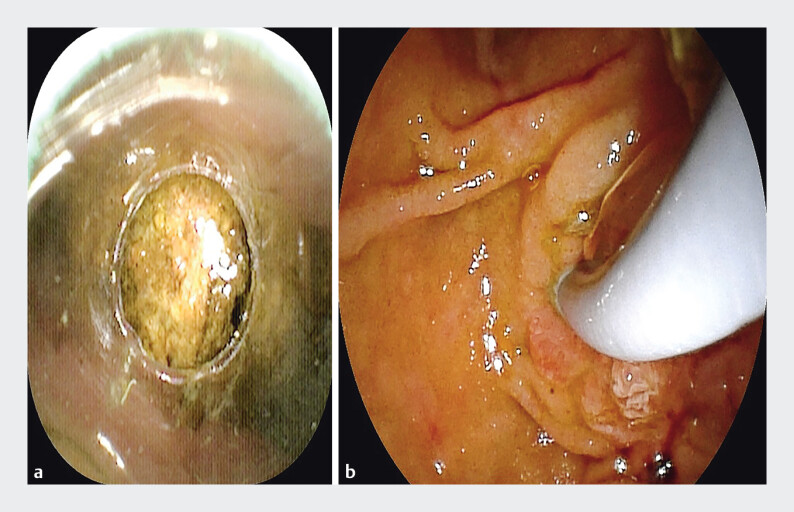
The transparent cylindrical cap provides clear visualisation of the calculus, including its precise location and dimensions.

**Fig. 3:**
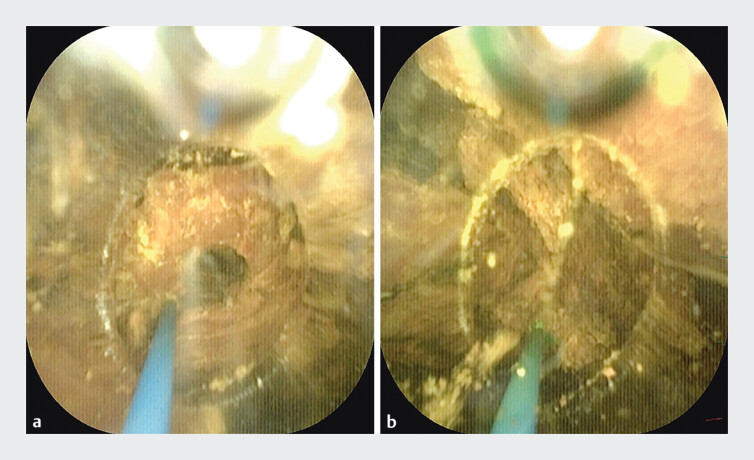
Transparent cap-assisted laser lithotripsy.

**Fig. 4:**
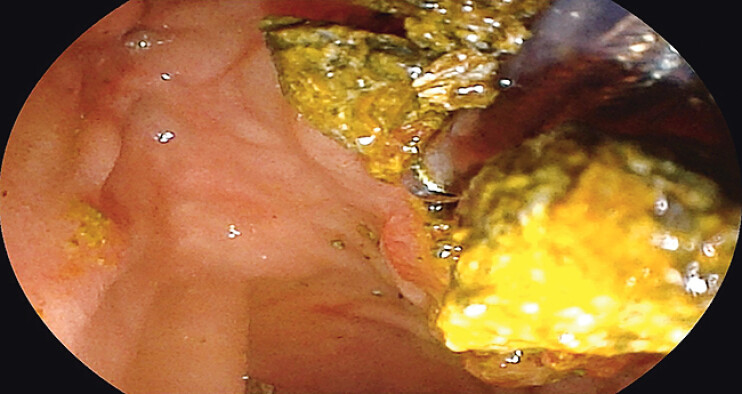
The basket extracted large stone fragments post-lithotripsy.

**Fig. 5:**
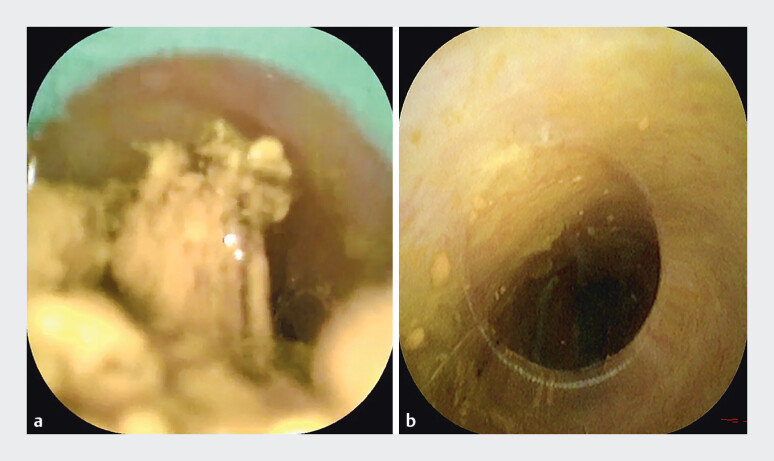
**a** The transparent cap facilitates aspiration of stone debris, **b** ensuring complete fragment removal.

Analysis of this case demonstrates that transparent cap-assisted biliary cholangioscopy offers many distinct advantages in treating biliary stones.Video 1

Analysis of this case demonstrates that transparent cap-assisted biliary cholangioscopy offers three distinct advantages in treating biliary stones: fixing stones enhances lithotripsy efficiency, protects the sub-mirror from damage, and shields the lithotripsy to provide a clear field of view.

Endoscopy_UCTN_Code_TTT_1AR_2AH

## Notes

Endoscopy E-Videos https://eref.thieme.de/e-videos
*E-Videos* is an open access online section of the journal *Endoscopy* , reporting on interesting cases and new techniques in gastroenterological endoscopy. All papers include a high-quality video and are published with a Creative Commons CC-BY license. Endoscopy E-Videos qualify for HINARI discounts and waivers and eligibility is automatically checked during the submission process. We grant 100% waivers to articles whose corresponding authors are based in Group A countries and 50% waivers to those who are based in Group B countries as classified by Research4Life (see: https://www.research4life.org/access/eligibility/ ). This section has its own submission website at https://mc.manuscriptcentral.com/e-videos .
